# Domestic violence and social norms in Norway and Brazil: A preliminary, qualitative study of attitudes and practices of health workers and criminal justice professionals

**DOI:** 10.1371/journal.pone.0243352

**Published:** 2020-12-04

**Authors:** Raquel Barbosa Miranda, Siri Lange

**Affiliations:** Department of Health Promotion and Development, Faculty of Psychology, University of Bergen, Bergen, Norway; University of Toronto, CANADA

## Abstract

**Background:**

Gender-based domestic violence has gained significant visibility in recent years and is currently considered a priority in the field of public health. This preliminary, qualitative study explores how social norms and professional regulations impact the attitudes and practices of health workers and criminal justice professionals regarding domestic violence against women in Brazil and Norway.

**Methods:**

A total of 16 semi-structured, in-depth interviews were conducted; eight in two different cities in Brazil, and eight in two different cities in Norway. In each country, four health workers and four criminal justice professionals were interviewed. We focused on the participants’ experiences with cases of domestic violence, their perceptions of their professional responsibilities, as well as the challenges they encounter. We analysed the transcribed interviews using a focused open coding process.

**Findings:**

The participants ranged in age from 32 to 59. All of them worked, with and without supervision, in cases involving domestic violence victims. In all four study locations, the professionals felt that they had not received enough training in how to handle domestic violence. Some medical doctors reported becoming personally detached over time, especially when the victims did not admit that their injuries were due to domestic violence. In the Brazilian cities, some professionals reported that women who were beaten by their partners were themselves responsible for the situation. This was not the case in the Norwegian cities. Both countries have laws and regulations that have been put in place to guide professionals who provide services to victims of domestic violence. For many reasons, professionals do not always follow these regulations. For the Norwegian health workers, confidentiality was an important factor explaining why they did not always report suspected domestic violence to the police. For the Brazilian health workers, the fear of having to testify in court, and thus potentially being vulnerable to violence themselves, was a factor that made some not want to involve the justice system. In both countries, the participating professionals reported the need for closer collaboration with social workers and mental health specialists, since domestic violence is closely related to both social norms in the communities and to individual psychological factors.

**Conclusion:**

Individual characteristics and experiences, the emphasis on confidentiality and the fear of repercussions may affect the way health and criminal justice workers perceive and deal with domestic violence cases. The findings in the study thus indicate that personal psychological factors and social norms concerning the acceptability of domestic violence are critical risk factors for women, and that a multi-professional approach is needed. The findings from this preliminary study can serve as background for larger and more comprehensive studies of how professionals handle cases of domestic violence.

## Introduction

Gender-based domestic violence has gained visibility and is currently considered an urgent matter in public health [1–4]. Domestic violence occurs globally and affects people of all ethnic groups and economic status, and women are the victims in most cases [2]. Domestic violence against women represents not only one of the most extreme expressions of gender inequality, as it is a violation of women’s human rights and fundamental freedom, but it is also a major obstacle to development [5].

Violence against women caused by an intimate partner is an important factor in harmful conditions for women’s health. The percentage of women who have reported being physically abused by an intimate partner varies from 15% to 71%, depending on the country [2]. Despite the alarming number of female victims of violence worldwide caused by a partner or ex-partner, most crimes against women are never registered. Domestic violence is difficult to measure with absolute precision due to numerous complications, including the social stigma that inhibits victims from disclosing their abuse [2, 4]. Health and criminal justice systems can play a fundamental role in preventing violence against women, helping victims to identify violence at an early stage, providing them with essential care, and referring women to proper and informed resources.

Domestic violence requires multidisciplinary coordination from bodies such as the criminal justice system (e.g., police officers, prosecutors, and the court system), the social system (e.g., legal aid, social services, and shelters), the community at large (e.g., neighbours, families, friends, schools, and churches), and health professionals (e.g., physicians, nurses, counsellors, and social workers) [6]. Nevertheless, coordination has not always been successful, as many health professionals do not recognise victims, and some police departments are not trained to properly deal with them and may humiliate the victims [7].

Female victims of domestic violence do not always denounce their aggressor; therefore, most remain anonymous, and the violence remains invisible [8]. However, when the victims suffer severe injuries that impair their physical health, they receive aid from health services. There are also cases when the violence does not cause severe physical harm, but where women turn to police stations in order to press charges against the perpetrator [9]. It is vital that public policymakers understand how professionals, who are part of the network of care for female victims, comprehend their role in health clinics and police stations.

Investigators in the field of domestic violence have recognised some barriers that affect the ability of both health professionals and patients to address the topic. Obstacles to physician inquiry include time restrictions, absence of training in dealing with domestic violence, embarrassment about this kind of harm, fear of upsetting patients, and feelings of ineffectiveness [10–12]. The reluctance of abused women to reveal violence to health or criminal justice professionals is based on fear of revenge by the abuser, embarrassment, humiliation, low self-esteem, and family devotion [13]. As the health community works to improve identification and intervention by healthcare professionals, lawmakers have created and enacted various pieces of legislation. Most countries require health workers to report all cases of injured patients to the police, and there are specific rules about including adult victims in care services [6]. Our hypothesis is that social norms, both in terms of prevailing gender roles and professional ethics, influence how professionals handle situations where they meet victims of domestic violence.

This study focuses on professionals’ experience with domestic violence in two cities in Brazil and two in Norway. Brazil and Norway are socio-economically and culturally very different countries. Despite having the largest economy in Latin America, Brazil has a high level of social inequality [14]. When it comes to domestic violence issues, one woman is killed every two hours, with an average of 4,500 women killed every year. The reported number of women murdered increased by 230% from 1980 to 2010 [15]. In 2006, the Maria da Penha Law was passed in Brazil. The law is an internationally lauded piece of legislation aimed at curbing domestic violence, which introduced special courts for victims. In the subsequent years (2006–2013), the number of reports on domestic abuse increased by 600%. This shows that the law encouraged victims to report assault and indicates that recent statistics do not necessarily signal higher levels of abuse against women, but instead might reflect higher levels of empowerment [16]. Despite the Maria da Penha Law, Brazil is still fighting against the high “hidden *cifra*”––hidden statistics, with reference to the high number of female victims who do not press charges.

Norway is a prosperous country recognised for its social welfare, gender equality, and social justice [17]. As for domestic violence, a nationwide, public educational campaign by Amnesty International Norway, carried out in 2005, showed that one in four Norwegian women has experienced domestic violence (physical or psychological), and every year, at least 20,000 women are exposed to (threats of) violence by someone with whom they share an intimate relationship. Less than half of these women contact public services for help [12, 18]. In the past few decades, the Norwegian government has taken many steps to fight domestic violence. These efforts have mainly been channelled through the government’s action plans to combat violence against women [19]. Gracia and Merlo (2016) have argued that a high prevalence of domestic violence rates in countries with a high level of gender equality, like Norway, might indicate that women in these countries feel more empowered and encouraged to talk about their victimisation [20]. The increase in violence may thus not reflect an actual higher prevalence, but rather higher levels of disclosure in comparison to less egalitarian countries.

This study aims to acquire preliminary insights into the way in which criminal justice professionals and healthcare workers handle cases of domestic violence, as well as their attitudes and practices in the contexts of Norway and Brazil. We explore how social norms and professional rules, as well as national regulations, impact professionals’ attitudes towards domestic violence through a small sample of participants in two cities in each country.

## Methods

### Research design

We selected a qualitative research design with the aim to acquire a preliminary understanding of the phenomenon through exploration rather than measurement. We adapted the questions developed by the Virtual Knowledge Centre to End Violence against Women and Girls, published by United Nations Women [21]. Although social norms were not explicitly mentioned in the interview guide, it was possible to identify their presence through the participants’ responses.

The interview guide focused on the participants’ experiences with cases of domestic violence in their professional work, and challenges in managing these cases (see Table 1). Each question was followed by probing to get further details about the participants’ experiences and perceptions.

**Table 1:** Interview guide.

1. Can you briefly describe your work and area of responsibility?
2. Do you treat victims who sustain injuries as a result of violence in the home? Is this frequent?
3. What are your primary concerns in serving these women?
4. Do you see injuries that you suspect are the result of violence in the home, but the woman gives another reason? How do you handle these cases?
5. If a woman tells you that her injuries were caused by violence in the home at the hands of her husband/boyfriend, what do you do? Do you document the injuries in a particular way? Do you refer her to other services?
6. How would you describe the level of coordination between hospitals and clinics and community groups, legal professionals, and the government?
7. Have you or your staff received any training related to documenting, for legal purposes, injuries resulting from violence in the home? What kind of training?
8. Has anyone close to you–family members, friends, or colleagues–ever experienced domestic violence?
9. Do you believe that having someone close with a history of domestic violence would affect the way you deal with these cases?

### Study area

We carried out the interviews in Brazil and in Norway. In each country, we chose one city with a large population and one city with a small population. The two cities in Brazil were in the south-eastern region, in the state of Espirito Santo, with a population of a little over 4 million. The two cities in Norway were in Vestland, the third largest county in the country, with approximately 637,000 inhabitants (more than 10% of Norway’s population).

### Participants

Using purposive sampling, we used a network of personal contacts who helped introduce us to criminal justice and health professionals in both countries. The selection of professionals privileged those directly involved in the process: medical doctors and nurses in the healthcare system, and police officers and prosecutors in the criminal justice system. The first author conducted 16 in-depth, semi-structured interviews (eight in Brazil and eight in Norway, with four health professionals and four criminal justice professionals in each country).

### Methods of data collection and accumulation

In Brazil, we conducted the interviews in Portuguese, while in Norway, English was used in five of the interviews and Norwegian in three. The interviews lasted between 30 to 40 minutes. Most lasted 35 minutes, mainly because health and criminal justice professionals had very busy workdays. The interviews took place in a private area in the chosen health clinics and police stations, or in another location in which the professional felt more comfortable. We audio-recorded all interviews. Interviews in Norwegian and Portuguese were transcribed in the original language, and then translated into English.

### Data management plan and analysis

We analysed the transcribed interviews using a focused open coding process [22]. We assigned in vivo codes (i.e., codes using the respondent’s words) to statements through a line-by-line, cross-interview analysis of the raw data using Atlas.ti, Version 8. The data analysis included the following steps: (1) *Understanding the transcriptions;* (2) *Coding data*; (3) *Identifying themes and linking them to the conceptual framework (social and professional norms)*.

## Ethical considerations

We invited all selected professionals to take part voluntarily in the study. We requested written consent from all participants, informing them that the interviews would be de-identified using a pseudonym. We received ethical clearance for this project from the Norwegian Social Science Data Services (NSD, #55285/2017) and from and the Federal University of Espirito Santo (UFES, #2213318/2017).

The participants were assigned pseudonyms to preserve their anonymity and confidentiality. An important ethical challenge was question eight, which asked the professionals about their personal experience with domestic violence *“Has anyone close to you–family members*, *friends*, *or colleagues–ever experienced domestic violence*?*”* This question was a very sensitive inquiry, and we assumed that some participants would not be comfortable answering this question. However, several professionals were very confident and open regarding this matter. In fact, this question proved to be an important door opener to the subject, because several of the respondents explained how these personal experiences had made them more conscious about the problem.

## Results

A total of 16 criminal justice and health professionals from Brazil and Norway participated in the study. They ranged in age from 32 to 59 and were equally distributed by gender in both countries. All of them were directly or indirectly working with domestic violence victims. The subsequent sections present the findings from the interviews organised along the following main themes: i) Health and criminal justice professionals’ own experiences, ii) Perceptions of domestic violence cases, iii) The role of education and training, iv) Regulation and laws, v) Confidentiality, vi) The importance of a multi-professional approach, and vii) Perceptions about barriers women face in terms of pressing charges and/or leaving the aggressor. The issue of social and professional norms is central in several of these themes.

## Health and criminal justice professionals’ own experiences

Professionals in both countries reported that they personally knew someone who has experienced domestic violence:

*A cousin who was living next door to me suffered violence at the hands of her husband*. *He hit her and it was a complicated situation*. *It was not easy to have it so close*.

(Ana, Brazil)

Like Ana from Brazil, Kristin from Norway stressed that even though she is a professional, it was very difficult to experience that someone close to her was a victim of domestic violence:

*It was a very bad experience and I needed to learn how to help her*. *It is more difficult when we are emotionally involved*.

(Kristin, Norway).

Answers varied on the question of whether having someone close to them who had experienced domestic violence would affect the way they handle similar issues, but no significant differences appeared between the countries. Fernando, a criminal justice professional from Brazil who has been working at a regular police station for 14 years, emphasised the importance of remaining impartial:

*We work here*, *right*? *We have to be neutral; we cannot take sides*, *and we always have to use the law*. *We have to be impartial*. *Mainly*, *we need to be fair and try to make the person who committed the crime pay for it*, *while also encouraging the victim to make that person take responsibility for the crime*, *and to realise that domestic violence is a crime*.

(Fernando, Brazil)

Birgitta, a police officer, and Arnt, a medical doctor from Norway, agreed about the importance of not becoming personally entangled in a case:

*Being involved*? *What is the premise on which the police have to act*? *A case is not truth*, *it is not a lie; a case…needs to be investigated*. *If we are not trained with the equity to identify*, *refer and investigate these cases*, *it may be that a previous experience with domestic violence can hinder our case management and referrals*. *We cannot allow this to happen*.

(Birgitta, Norway)

Arnt stressed that he had been trained to avoid becoming involved, because doing so could interfere with his professional approach.

*I was trained to not allow this [case involvement] to happen*. *We need to be professional to provide good care*.

(Arnt, Norway)

There were no significant differences between the countries. The aim of being professional and not getting personally involved in a case of domestic violence was considered important in both professional groups. This demonstrated that professional concepts in healthcare and criminal justice were similar irrespective of the country.

### Perceptions of domestic violence cases

According to some participants, violence against women is a historical problem rooted in the following social mentality: a mindset that accepts the superiority of men and imposes a submissive role for women, the subordination of women, and men’s control over women’s lives and their decisions. This problem is aggravated by women’s social, cultural, and economic conditions. Some participants in both countries recognised and linked patriarchy to violence against women, while others seemed to ignore this link. Through the interviews, we noticed the extent to which patriarchy is rooted in society in such a way that some forms of violent behaviour toward women are considered normal, or at least justifiable. Sofia, a Brazilian sheriff who previously worked at a women’s police station and dealt with many domestic violence cases, expressed the belief that males should not take all the blame for patriarchal attitudes and domestic violence:

*It’s not a matter of only educating men*. *Once I dealt with a case of violence against a woman who had been beaten since the third day of her marriage*. *She was married for 25 years*, *and her mother-in-law—a woman*!*—told her son to beat her; not only that*, *she told him to beat her in the intimate areas because she would be ashamed to show her body to someone*. *That way*, *he would not be denounced*. *It was an act on the part of a woman—but she is just a regular person who was raised in the same society as men*. *There are men and women with deeply sexist behaviour*, *because the society they live in is the same*. *We are all villains and victims of ourselves*. *Men are raised by women*. *You see*?

(Sofia, Brazil)

What this participant told about women with sexist behaviour was, unfortunately, exemplified in another interview with a professional from Brazil’s criminal justice system. Maria works directly with victims, who seek free assistance at the police station:

*Many women come back here to withdraw charges against their husbands*. *Once a woman came here and told me she wanted to withdraw the charges against her husband because she believed she was the one to blame for what he had done to her*. *I asked her to explain the case better*. *She said her husband had lost his job*, *and because of that*, *he started drinking a lot every night and coming home very late*. *She said one night she had had enough and locked the house*, *not letting him in*. *I asked her why she would do something like that*. *I asked her if she understood that she really did contribute to the aggression*.

(Maria, Brazil)

In Norway, none of the interviewed police officers expressed sexist attitudes, but Dagfinn, a police officer, showed concern for the patriarchal and sexist mentality of some professionals in the criminal justice system:

*Look*, *to me*, *the crime of violence against women is very serious*. *It affects me a lot because I have had experiences like this in my family*, *so maybe this has consequences for my analysis*. *I am very strict in cases of domestic violence*. *However*, *I will tell you that some cops and judges think differently*. *The trend of placing the blame on the woman remains*, *and/or that the woman causes the events (…) Perhaps this also happens because it is very common for female victims*, *who can be given protective measures*, *to call their husbands to encourage them to return home*. *That is*, *women ask for protective measures and afterwards ask their husbands to come back home*.

(Dagfinn, Norway)

Fredrik, another Norwegian police officer, similarly reported that women often do not press charges against the aggressor, even after they called for help. The aggression seems to be justified by alcohol consumption:

*We often get calls regarding domestic violence during the weekends when people have been consuming alcohol in excess*. *It’s common that the women say they had enough and want to move out of the house*. *But when Monday comes and they are sober*, *they give up and do not want to cooperate with the police anymore*.

*(Fredrik*, *Norway)*

We noticed some similarities between Brazil and Norway regarding male-dominant behaviour. However, professionals in Norway claim that things are changing and have a more proactive approach to the problem. Unlike some of the participants in Brazil, none of the Norwegian professionals believed that women were responsible in some way for the aggression. However, male criminal justice professionals from both countries were indignant about the fact that women themselves violate restraining orders. This was mentioned as a dangerous behavior and seen as a barrier to police work.

### The role of education and training

Regarding the training to prepare the interviewed professionals to deal with cases of domestic violence, we noted that there is no specific protocol. Below, we describe the different perspectives of two Brazilian criminal justice professionals with regard to training:

*We have received several training sessions*. *We have our own training regarding the victims’ approach*: *(1) identify cases of violence; (2) make referrals; and (3) know the health system*. *You know that many cops are still unaware of how care for women in health units works*. *Furthermore*, *in partnership with regional prosecutors*, *the Center for Confronting Domestic and Family Violence lies at the core of domestic violence*, *and provides training throughout the state*, *both for civilian and military police*.

(Maria, Brazil)

Even though both professionals work in the same city, one of them received training, while the other did not:

*I never received any training on domestic violence*. *It is really up to the sheriff or police officer*, *or to the professional in charge*, *to discuss and decide what to do*. *We need to seek the most current information regarding this matter and talk to other colleagues*, *because there is no systematic training for how to deal with this type of crime or this kind of victim*.

(Fernando, Brazil)

As for the health professionals, almost all of them declared that they had had lectures regarding violence when at university. However, only a few of them were trained to deal with domestic violence cases, to be able to address the violence with their patients. In the words of Pedro from Brazil:

*I have been trained to deal with domestic violence cases when I started to work in the Family Health Program*. *They taught us to recognise and face complex situations (…) and to deal with them*. *It [the course] was very important for me as a professional*!

(Pedro, Brazil)

Hanna, a medical doctor from Norway, received training at medical school as well as brief courses afterwards:

*I received training in my previous work*. *I participated in local courses in the hospital*, *as well as through medical education*. *I was trained to take care of the physical injuries caused by violence and how to behave in these situations*.

(Hanna, Norway)

Fredrik, a police officer from Norway, also emphasised the value of several rounds of training:

*I received training in Oslo [the capital city]*. *I also took local courses in the police force*, *as well as during my police education*, *when I was trained to take care of the legal aspects of injuries caused by domestic violence and how to proceed in such circumstances*.

(Fredrik, Norway)

Some of the professionals who did not receive any follow-up training reported that they have had some challenging episodes regarding domestic violence cases:

*Of course*, *we all go through basic courses at university where we are taught how to recognise violence against women and kids*. *To be honest with you*, *I have never taken another course on that after I graduated from medical school*. *It would have been interesting to have a course on that*. *It can be very challenging sometimes*.

*(Kristin*, *Norway)*

There was no significant difference between Brazil and Norway in terms of training professionals to deal with domestic violence, despite the fact that health and criminal justice professionals comprise the frontline social institution for dealing with domestic violence and are often the desired point of disclosure for victims. We noted dissimilar reports and realised that training is not offered on a regular basis in Brazil or Norway, especially not for professionals who work in smaller municipalities.

### Regulations and laws

Both countries have laws providing guidance on services related to domestic violence cases. For many reasons, professionals do not always follow these rules, but the situation is more complex in Brazil, where the structure of services faces several challenges.

In both Brazil and Norway, the law guarantees that violence against women is a crime that does not require one’s permission or representation to enable police to enforce the law and criminally charge the offender. When a woman suffers violence by her partner, that action becomes the responsibility of the State, and the police have the duty to open a case and assure victims protection irrespective of whether they want to press charges. Fredrik, a Norwegian police officer, explained it in this way:

*In the cases where we are sure that the woman is being abused in her home or relationship*, *according to Norwegian law*, *the police must open a case irrespective of the victim’s will*. *It applies for all family cases related to physical violence*. *But the cases where we can act without the victim’s cooperation are in the minority*.

*(Fredrik*, *Norway)*

In Brazil, a high percentage of women still fear pressing charges against the aggressor and making the act of violence public. This was of concern to criminal justice professionals:

*We currently have the so-called hidden cifra*. *What is this*? *It means that many women do not turn to police stations or public services for help or to report their problems*. *This is because they do not think they will receive assistance from public services*. *They are afraid of being re-victimised*, *of being blamed for the event*. *This is very common*. *Today*, *20% to 30% of women do not use public services for their protection because they are afraid*. *We realize that this number is much higher than statistics reveal*.

(Sofia, Brazil)

It was possible to perceive the connection between social norms and the importance they ascribe to regulations in professionals’ statements from both countries. Thiago, from the Brazilian criminal justice system, linked domestic violence with gender issues:

*We have a much more serious problem than statistics show*. *There is no doubt about that*. *How many women are beaten daily*? *Are they not violated in terms of their morals and heritage*? *These women do not turn to police units due to the challenges of recording an incident*, *which in turn is due to the difficulty of obtaining protective measures because of swift hearings*. *Today we still have a very serious problem regarding gender violence*. *This worries us*.

(Thiago, Brazil)

Fredrik, who has worked in the same police station in Norway for 15 years, demonstrated an impressive knowledge about Norwegian legislation for domestic violence cases:

*In 2010*, *the government approved a new piece of legislation that includes a statutory*, *municipal responsibility to provide shelter services to the local population*, *regardless of gender*. *(…) I believe that the law went into effect in 2010*. *The main purpose for providing a statutory shelter service is to show that public authorities are responsible for safeguarding people*.

(Fredrik, Norway)

Both countries have regulations on domestic violence assistance, and criminal justice professionals seem to value and follow them. The situation seems more complex for health professionals because they need to find a balance between following the ethical medical code and ensuring patient safety. Health professionals also face the difficulty of identifying violence suffered by the patient when this is not obvious, while police usually help women who are somehow ready to reveal their situation. As for criminal justice professionals, the ones from Brazil have to work with the fact that some victims might be sceptical to approach them because of previous cases of police misconduct toward women based on patriarchal believes.

### Confidentiality

Many of the participants raised the issue of confidentiality. This was linked both to the victim’s right to confidentiality (Norway) and to the concern for one’s own safety (Brazil).

Pedro, a gynaecologist who has been working in a public hospital in Brazil since 1998, reported that he has never seen or suspected that any of his patients had been through a case of domestic violence. We found such testimony very shocking, considering that the hospital where Pedro works–which is also where we held the interview–is located in a poor part of the city where there are many reports of domestic violence. We found it quite unlikely that the women who sought help at this hospital–over a period of almost twenty years–never showed up with any signs of physical or psychological aggression. During the interview, however, it became clear why the doctor had not been so attentive to the possible assaults suffered by his patients. The apparent “lack of sensibility” in this case appears to be caused by fear of exposure, caused by a failure in the system:

*(…) when we [health professionals] make the notification*, *we already know who will provide answers in the justice system (…)*, *it will not be the hospital*, *municipality or the government*. *Rather*, *the physician will do it*. *Thus*, *the one who will be exposed and will need to participate in the situation is each one of us [professionals] individually (…)*. *We become very vulnerable; we do not know the character of the alleged aggressors*. *Not just us*, *but our families*, *too*, *you see*? *Our name appears publicly during the process; the entire population has access to our names*, *our work schedules*, *and on the internet you can find the rest of the information about us*. *I think our team fears that*.

(Pedro, Brazil)

In Norway, the participants were not afraid of retaliation from the aggressor, as in Brazil, but healthcare professionals feared becoming involved in a patient’s private life beyond what is considered acceptable in society, as well as of breaching doctor-patient confidentiality. Hanna, a doctor from Norway, talked about the difficulties involved in dealing with the problem, despite the fact that she has been working in the same clinic for 25 years. Sometimes she feels there is something wrong; she tries to help but she cannot do anything if the woman does not want to talk about the situation:

*You can never know for sure*, *because if you ask them*, *they say no*. *Yet you kind of feel that there is something there*. *You can tell that these injuries don’t result from falling down the stairs*. *For instance*, *if they have bruises internally*, *you don’t get that if you injure yourself on the stairs or something like that*. *It’s very difficult to get bruises internally unless somebody held you down or did something bad to you*. *So of course*, *with these cases–it kind of makes you think*, *right*? *If you ask them*, *they say no*, *but if you ask a bit more*, *you gradually see that they start shaking*. *However*, *at the same time*, *I think it’s important to remember that we want to help*, *we want to do something about it*. *But it is not about us*, *it is about them*, *and if they don’t want help*, *we have no business saying anything*.

(Hanna, Norway)

Doctor-patient confidentiality was mentioned several times by both Brazilian and Norwegian health professionals as the main reason why they sometimes do not report cases. They were consistent in underscoring the importance of respecting the patient’s will:

*Most of the time they [women] deny it*, *no matter how much you realise it*. *You realise that the person is not telling you the truth*, *but you respect them because you will not be able to intervene*. *I’m not going to tell her [a woman] that she’s lying*, *that her injuries actually resulted from getting beaten up*. *I know she’s lying*, *but I cannot tell her she’s lying*. *We need to respect the other person because I cannot distort information that the patient is giving me*. *I will not confront her*, *right*? *Yet I realise that the story is false*. (…) *What prevents her from disclosing is the fear that the violence will get worse*, *and this can get to a point of no return*. *Because of that*, *I always respect the patient’s wish to report the incident or not report it*.

(Elizabete, Brazil)

In contrast, criminal justice professionals in both countries insisted that all cases should be reported to the police station as required by law:

*Domestic violence is a crime*, *and it is the police’s duty to investigate it*, *regardless of the woman’s will*. *In most instances*, *health professionals do not call the police*.

(Fernando, Brazil)

Participants from the police/law sector argued that if patients are at risk, health professionals should not avoid becoming involved, despite the ethical guidelines on doctor-patient confidentiality:

*Confidentiality between the doctor and patient is not supposed to be that strict anymore*. *If the doctor sees that the patient is facing some kind of risk*, *they have to notify the police immediately*. *Cooperation with hospitals has become much better now*.

(Dagfinn, Norway)

In addition to the issue of confidentiality and fear of repercussions, some of the medical doctors explained that their personal engagement with victims of domestic violence had changed with time:

*To be honest*, *I think when I began my studies in Oslo as a medical student–I don’t know if I was naïve or hadn’t been exposed to this before–but I was more concerned about the psychological part*. *I was worried about the children of these women and their security*. *A lot of women showed up with severe injuries*. *I asked them if they were going to file a report with the police against the aggressor*, *and they would say no*. *My primary concern was to try to make them understand that there is an end to this and we can try to help them (…)*, *but I think that nowadays*, *I kind of stopped caring so much in this sense*. *Like*, *I think it took me a while to understand that it is not my life*. *You just see it so much*. *More and more*, *I feel like I am just treating an object*, *so my primary concerns are the physical injuries*.

(Kristin, Norway)

In addition to the fear of violating patients’ privacy then, we observed that some doctors developed a sense of detachment over time.

### A multi-professional approach

Some interview questions helped us to determine the relationship between the approaches of professionals, institutions, and violence. These questions covered how to document injuries and refer the victim to other services; the level of coordination between hospitals/clinics and criminal justice professionals; and whether the institution provided specific training related to documenting injuries resulting from violence in the home, for legal purposes. We perceived that integration between the different institutions involved in women’s care is complex due to several aspects.

The link between criminal justice and health professionals is not as strong as it should be, and the fact that women count on health professionals for confidentiality makes the situation even more challenging. In the words of one of the Norwegian police officers:

*The cooperation between hospitals or clinics and police stations is very complex*! *There is no perfection*. *It is public knowledge that a gap remains regarding victim care (…)*

(Einar, Norway)

Julia, a Brazilian nurse, explained that they send the information that they are supposed to send to the police—if the patient allows them:

*What is the level of collaboration between the hospital and police*? *I provide them with the written medical record if the patient gives consent*. *The government does not cooperate with me*. *I collaborate with the police office; there is a standing agreement for me to obtain help from police when I need it and when they need me*, *if the patient agrees*.

(Julia, Brazil)

Health and criminal justice professionals in both countries attributed the limitations of their work to a lack of support from mental health professionals and social workers:

*It [domestic violence] is a complicated topic and…[that is] why we need a psychologist to help us conduct these cases*.

(Dagfinn, Norway)

In Brazil as well, professionals expressed concern over the lack of psychological professionals, which was thought to be essential to complement multidisciplinary work.

*The specialised women’s police stations (…) have social workers as part of their structure–but they do not yet have psychologists*. *They usually do this work to receive the women in the first place*, *and if it is necessary*, *to send them somewhere for more in-depth psychological assistance*. *This is the job of social workers*, *right*? *I am very happy to count on them to help me*!

(Thiago, Brazil)

In a Brazilian clinic where they added a social worker to the multi-professional team, the doctor was relieved to know she could count on this professional.

*We have some cases where the women are very emotional*. *Domestic violence really affects them emotionally*, *and this is challenging to deal with*. *We send them to the social worker or the psychologist to be listened to*, *right*? *We have an excellent social worker working with us*, *thank God*! *It was a wonderful acquisition for our clinic (…) She solves the emotional part of the problem*!

(Ana, Brazil)

Interestingly, most of the professionals from both countries felt they were not properly trained to take the lead in terms of the social and psychological aspects of domestic violence:

*I do not always feel prepared to take care of psychological problems*. *We need help from professionals*. *Some nurses are good at that though*.

(Agnes, Norway)

Hanna reported that time constraints also play a role:

*My primary concern as a health professional would be the physical injuries*, *because the number of domestic violence victims is increasing*, *and we don’t have time to provide psychological support to all of them*.

(Hanna, Norway)

Both health and criminal justice professionals from both countries talked about the difficulties regarding the social and psychological aspects. The need for extra support was unanimous. They are trained and used to working on concrete problems. They feel insecure (or are too busy) to spend time on emotional issues.

### Perceptions about barriers women face in terms of pressing charges and/or leaving the aggressor

In Brazil, when speaking about the barriers women face in terms of pressing charges and/or leaving the aggressor, the importance of women’s economic independence is underscored as an element of a possible transformation in the male-female relationship, the end goal being for victims to achieve positions of equality and freedom. Such positions allow them to build new partnerships to overcome violence.

Thiago, a Brazilian police officer, talked about women’s circumstances from the angle of disadvantages, and affirmed that financial issues play an important role in women’s staying in violent relationships:

*We have substantive indications that women depend on their husbands*, *and generally*, *we also see that many victims have many children*. *(…) I am sure there are other things that keep a woman in a toxic relationship*. *However*, *I believe that being financially dependent on one’s husband is the biggest factor*.

(Thiago, Brazil)

In contrast to the almost unanimous belief that what keeps a woman in a violent relationship in Brazil is economic dependence, a participant from the Brazilian criminal justice system believed that what kept a woman in a violent relationship was not only economic dependence, but also included psychological dependency:

*Many people think that financial dependence is the biggest factor*, *but financial dependence happens because of psychological dependence*, *and psychological dependence does not happen overnight*. *Everyone has a hard time understanding*, *but why does she submit to her husband*? *This is a construction*, *it starts with a small amount of violence until it reaches a level whereby the victim can no longer leave the relationship*, *and both aggressor and victim become ill*.

(Maria, Brazil)

One of the Norwegian criminal justice professionals also reported psychological dependence as a barrier for women to leave their abusers:

*My concern is that they [women] usually go back to the same man and the abuse continues*. *Some women do not have the courage to make the decision to leave the aggressor and frequently go back to those men*. *They feel they might regret abandoning their home*.

(Birgitta, Norway)

In some cases, professionals thought that the woman’s dependence on her partner was linked not only to psychological issues, but also to social norms about maintaining the family structure. They care about having a family for their children:

*I am concerned about doing my job and ending the abuse these women suffer*. *The problem is that when we have one case of family abuse per month at the police station*, *there are at least ten other cases that we do not know about*. *When they [the women] come to press charges*, *it is because the violence has happened many times already*. *For the sake of the family*, *women here decide to forgive their partners*.

(Dagfinn, Norway)

In Brazil, the importance of women’s economic and psychological independence was reported to be a central factor in the process of breaking free from domestic violence. In Norway, where the level of social security/economic support from the state is relatively good, participants reported psychological dependence and social norms as the main barriers to ending violence; for example, psychological dependence and social norms caused women to return to the same man for the sake of the family–even after being abused. Some women do not want to break up the family and expose their children to divorce.

## Discussion

All the interviewed criminal justice and health professionals report that domestic violence is a very challenging and complex issue to work with. Criminal justice professionals have been criticised for assigning low priority to domestic violence cases and for sometimes blaming the victims [9]. One reason may be that domestic violence offences relatively frequently recur in the courts [23] and that some women continue living with partners who mistreat them. To place the responsibility of the violence with the victim [16] is relatively common, and some of our participants in Brazil expressed such attitudes. This is linked to the fact that the idea of *machismo* (or the strong and powerful “macho man”) is still prevalent in Brazilian society. Attitudes towards domestic violence are closely linked to perceptions of gender roles, and in some contexts and under certain circumstances, women may justify physical violence on the part of their husband [24].

There are some notable differences between Brazil and Norway when it comes to social norms and factors that may keep health workers from reporting a case of domestic violence, but in both countries, professionals emphasised that they try to conduct cases with impartiality and to follow protocol. They also pointed to the importance of not allowing personal experiences to interfere with their jobs. Health professionals are expected to act in their patients’ best interests, even when those interests might conflict with their own [10–12].

Unlike the USA [25], both Brazil and Norway have laws stating that all cases of domestic violence should be investigated and reported to the police despite the victims’ will [26–28]. Health professionals are thus obliged by law to report cases, but do not always do so. Despite the significance of legislation, health professionals in both countries mentioned doctor-patient confidentiality as one of the barriers to working on domestic violence cases, and as the reason for not reporting cases to authorities. A good physician-patient relationship includes understanding, confidence, encouragement, reassurance, and confidentiality, which can help women with violent experiences overcome obstacles and feel safe enough to talk about the problem with the relevant authorities [29]. Brazil has protective laws [26], but before women press charges against their aggressors, they need to be convinced that legislation will be effective. One of our participants however, feared that her female patients could potentially suffer even more if she encouraged them to report the case.

There is a general belief that domestic violence is a private issue and can only be resolved in a private setting. The problem is that in many cases, health professionals and patients share this belief. An important problem disclosed by health professionals, mostly in Norway, but also in Brazil, was the fear of interfering in someone’s private life when help was not requested.

One of the Brazilian doctors who participated in our study feared violence if he were to testify in domestic violence cases in court. Anecdotal evidence from the USA shows that some police officers believe that domestic violence investigations differ from all other types of police investigation, as officers face the threat of potential violence not only from the offenders, but also from victims [30]. Further research is needed to address how professionals who are asked to testify in court cases can do so without becoming personally vulnerable.

One of the participants in our study, a sheriff from Brazil, stated that many women call the police about a case of violence, but when the police go to the crime scene and take both offender and victim to the police station, the woman refuses to press charges. A police officer from Norway who participated in our study reported many cases where alcohol consumption was involved when the call to police was made. Once things calmed down and both parties were sober, the woman did not want to press charges anymore, which made the situation challenging. The social acceptance of immoderate alcohol consumption during weekends in Norway may by some be seen as justifying violent behaviour [31]. One of the Norwegian criminal justice professionals reported the challenges of collecting information and solving cases, despite the existence of crisis centres and the implementation of the spousal assault risk assessment tool (SARA).

### Training

In general, our findings show that most of the interviewed professionals seem to be doing the best that they can within their responsibilities; however, they did not feel that they were adequately trained to deal with domestic violence cases. Some of the participants had never been trained to deal with this issue at all; others had been trained as part of their education but had never received any follow-up training. Further education and training of healthcare and criminal justice professions in identifying and addressing domestic violence has been proposed as one way to resolve the many related obstacles. This could improve health outcomes for victims [11, 20]. Domestic violence training and support programs for primary care have been predicted to be cost-effective from the angle of community [32, 33]. The importance of adequate training is linked with decreasing the stigma associated with domestic violence.

Our findings revealed that criminal justice professionals had a sense of responsibility toward the effects of the law, as well as the desire to resolve cases. Despite viewing domestic violence cases and their victims in a critical light, research has shown that judges favour increased training in arbitrating domestic violence cases [9]. In this study, the criminal justice professionals from both countries complained about the lack of training and not feeling prepared to deal with the social or psychological aspects of domestic violence cases.

Regarding the health system, a major barrier to asking questions in domestic violence cases includes healthcare professionals’ belief that by screening for domestic violence, they will become involved in a private and complicated situation that they are not sure they are prepared to handle, since they feel they have not been satisfactorily trained [34]. Some health professionals that were interviewed thought they should not get involved in cases of violence because they do not have the proper instruments at health facilities to enable them to deal with such a complex situation. Many mentioned the duty of confidentiality between doctor and patient as preventing them from assisting the patient beyond physical health care, such as contacting the police.

Regarding the influence of institutions’ regulations on the attitudes and practices of health and criminal justice professionals, they find ethical codes and institutions’ regulations to be very important. Health professionals are very concerned about ethical codes, while criminal justice professionals are concerned about laws and regulations. Rather than commanding specific actions, a code should explain the ethical environment for the provision of healthcare and reproduce its character and general approach [35, 36]. These concepts emerged very frequently in our interviews. The notion of a professional ethical code was highly present when the health professionals talked about the importance of confidentiality. They described their worry about confidentiality as a barrier to helping patients end physical abuse.

Interestingly, when asked about the link between hospitals and police stations, the criminal justice professionals recognised confidentiality as a problem that, in many cases, would expose the woman to further risk, which interferes in the police’s capability to help and protect the victim. This is a real possibility. However, it is important to consider cases where physician involvement can expose the victim even more. In addition to concerns about confidentiality and the presence of professional codes [35, 36], there are also government regulations on caring for victims that professionals are to adhere to.

Whether they are working in the criminal, civil, or family courts, criminal justice professionals have fundamental functions in domestic violence cases. These functions often meet conventional expectations that society has for them. For example, criminal justice professionals might also be expected to provide social services for female victims, despite the fact that they are not trained to do so and do not see themselves playing the roles of social workers [6, 9]. Our study found that both health and law professionals would appreciate closer collaboration with social workers.

### The need for a multidisciplinary team

Some of the health workers interviewed for this study argue that they limit themselves to concentrating strictly on the physical aspects of health–a finding that has been reported elsewhere [11, 37]. This does not always match the expectations of the women, who envision comprehensive services, versus merely addressing symptomatic problems [10, 11]. Consequently, many women do not disclose abuse and many health professionals do not ask about violence during clinical consultations. This may be because the period of time available for the appointment may be very short, or simply because the matter is difficult to deal with.

In Brazil, the fifth article of the Maria da Penha law that deals with domestic violence foresees the formation of a multidisciplinary care team, to consist of professionals that specialise in the psychosocial, legal, and healthcare fields [26]. In Norway, a multi-professional approach is also recommended in cases of domestic violence. Health and criminal justice professionals can refer victims to social and psychological resources using the *krisesenter* (crisis centre) facilities [27]. Yet as we observed in the interviews, there are still some gaps in the connections among them.

Besides having an interdisciplinary team working on domestic violence cases, it is also important to build a network. Networking among relevant professionals and institutions is an appropriate way to improve victims’ circumstances and the efficiency of the approach [21, 33]. Exchanging knowledge and experience, being well informed of what others are working on, having direct links to other organisations, and knowing about training possibilities are great advantages of these cooperative networks.

A frequent comment by the participants in this study was the victims’ need for psychological support. The interviewed professionals who could count on social and psychological support in their workplaces reported more safety while managing cases. On the other hand, those who did not have this kind of support described difficulties. Their remarks emphasize the importance of having a multidisciplinary team that should comprise several professionals from different specialities who act as a group. Moreover, there is a need for a good relationship between the team members and recognition of the patients’ situation in both medical, social and psychological terms [6, 10].

Professionals who work with the phenomenon of violence must position themselves as facilitators of therapy, building strategies that contemplate and respect the social context and singularities of victims. To boost this process, it is necessary to approach the realities experienced by victims and to make the conflicts presented during the complaints visible, from a multidisciplinary perspective [6, 9, 10]. Measures should be prioritized to support women in situations of violence, a strengthening of legal measures, strategies to empower women and improved access to multi-professional care (e.g., the psychological, social, legal, and health fields).

## Conclusions

Over the past few decades, there has been a significant development in adopting international policies to address domestic violence in Brazil and Norway. These countries’ governments have passed laws to reduce domestic violence, to investigate and prosecute cases and to punish the perpetrators. However, there is still a need to improve the quantity and quality of training for criminal justice professionals and health workers on how to handle cases of domestic violence. A central finding of this preliminary study is that a central professional norm for health workers–confidentiality–conflicts with the legal requirement to report cases of domestic violence. Further research is needed to ascertain how health workers handle this dilemma.

## Limitations

This is a preliminary study with a low number of participants within each category. This was due to limitations in terms of time and resources. However, we think that our findings demonstrate the importance of including the perspectives of both criminal justice and health professionals when we study domestic violence. We also think that including participants from both Brazilian and Norwegian cities was important to show that many of the challenges that professionals face regarding domestic violence are the same even in countries that are very different culturally and in terms of the level of social security that is offered by the state. The interviews were relatively short, lasting for 30–40 minutes. The reason for this was that it was hard to recruit participants due to their very busy work schedules. Upon recruitment, we therefore promised the participants that we would not take more than a maximum of 45 minutes of their time. Social desirability may also have played a role, due to the overall tendency of people to give socially acceptable answers in face-to-face interviews.

## Supporting information

S1 File Interview guide in English. (DOCX)

S2 File Interview guide in Norwegian. (DOCX)

S3 File Interview guide in Portuguese. (DOCX)
