# Supplementary material for: Domestic violence and social norms in Norway and Brazil: A preliminary, qualitative study of attitudes and practices of health workers and criminal justice professionals
Source: PLoS One. 2020 Dec 4;15(12):e0243352. doi: 10.1371/journal.pone.0243352 (PMC7717503; doi:10.1371/journal.pone.0243352)
Supplement: S1 File — (DOCX) [file pone.0243352.s001.docx]

**Interview guide, English**

The interview guide was adapted from questions developed by the Virtual Knowledge Centre to End Violence against Women and Girls (Available from http://www.endvawnow.org/en/articles/863questionsformedicalprofessionals.html)

**Domestic violence and social norms: Brazilian and Norwegian criminal justice and health workers’ attitudes and practices**

(1) Can you briefly describe your work and area of responsibility?

(2) Do you treat victims who sustain injuries as a result of violence in the home? Is it frequent?

(3) What are your primary concerns in serving these women?

(4) Do you see injuries that you suspect are the result of violence in the home, but are explained by the woman by another reason? How do you handle these cases?

(5) If a woman tells you that her injuries are the result of violence in the home by her husband/boyfriend, what do you do? Do you document the injuries in a particular way? Do you refer her to other services?

(6) How would you describe the level of coordination between hospital or clinic and community groups, legal professionals, or the government?

(7) Have you or your staff received any training related to documenting, for legal purposes, injuries that are the result of violence in the home? What kind of training?

(8) Has anyone close to you – family, friend or colleague – ever experienced domestic violence?

(9) Do you believe that having someone close with a history of domestic violence would affect the way you deal with these cases?
