# Supplementary material for: Domestic violence and social norms in Norway and Brazil: A preliminary, qualitative study of attitudes and practices of health workers and criminal justice professionals
Source: PLoS One. 2020 Dec 4;15(12):e0243352. doi: 10.1371/journal.pone.0243352 (PMC7717503; doi:10.1371/journal.pone.0243352)
Supplement: S2 File — (DOCX) [file pone.0243352.s002.docx]

**Interview guide, Norwegian**

The interview guide was adapted from questions developed by the Virtual Knowledge Centre to End Violence against Women and Girls (Available from http://www.endvawnow.org/en/articles/863questionsformedicalprofessionals.html)

**Vold i hjemmet og sosiale normer: Holdninger og praksiser hos Brasiliansk og norsk juridisk personell og helsearbeidere** [Domestic violence and social norms: Brazilian and Norwegian legal and health workers’ attitudes and practices]

(1) Kan du kort beskrive arbeidet ditt og ansvarsområdet ditt?

(2) Mottar du ofre som du tror har skader som følge av vold i hjemmet? Hvis ja, skjer det hyppig?

(3) Hva er dine primære bekymringer i å betjene disse kvinnene?

(4) Ser du skader som du mistenker er et resultat av vold i hjemmet, men der kvinnen oppgir en annen grunn? Hvis ja, hvordan håndterer du disse sakene?

(5). Hvis en kvinne forteller deg at skadene hennes er et resultat av vold i hjemmet av ektefellen/kjæresten, hva gjør du? Dokumenterer du skader på en bestemt måte? Henviser du henne til andre tjenester?

(6) Hvordan vil du beskrive samarbeidsnivået mellom sykehus eller klinikk og samfunnsgrupper, advokater eller regjeringen?

(7) Har du eller din medarbeider fått opplæring i forbindelse med dokumentasjon av legemsskader som skyldes vold i hjemmet? Hva slags trening?

(8) Har noen i nærheten av deg - familie, venn eller kollega - noen gang opplevd vold i hjemmet?

(9) Tror du at det å ha en nær person med en historie med vold i hjemmet ville ha påvirket måten du håndterer disse sakene?
