# Supplementary material for: Domestic violence and social norms in Norway and Brazil: A preliminary, qualitative study of attitudes and practices of health workers and criminal justice professionals
Source: PLoS One. 2020 Dec 4;15(12):e0243352. doi: 10.1371/journal.pone.0243352 (PMC7717503; doi:10.1371/journal.pone.0243352)
Supplement: S3 File — (DOCX) [file pone.0243352.s003.docx]

**Interview guide, Portuguese**

The interview guide was adapted from questions developed by the Virtual Knowledge Centre to End Violence against Women and Girls (Available from http://www.endvawnow.org/en/articles/863questionsformedicalprofessionals.html)

**Violência Doméstica e Normas Sociais: As Atitudes e Práticas dos Trabalhadores Jurídicos e de Saúde do Brasil e da Noruega** [Domestic violence and social norms: Brazilian and Norwegian legal and health workers’ attitudes and practices]

(1) Você pode descrever brevemente o seu trabalho e área de responsabilidade?

(2) Você trata as vítimas que sofrem ferimentos como resultado da violência em casa? É frequente?

(3) Quais são suas principais preocupações ao atender essas mulheres?

(4) Você vê lesões que você suspeita serem o resultado da violência em casa, mas são explicadas pela mulher por outro motivo? Como você lida com esses casos?

(5) Se uma mulher lhe disser que seus ferimentos são o resultado de violência em casa por seu marido / namorado, o que você faz? Você documenta as lesões de uma maneira particular? Você a encaminha para outros serviços?

(6) Como descreveria o nível de coordenação entre o hospital ou a clínica e os grupos comunitários, os profissionais legais ou o governo?

(7) Você ou a sua equipe receberam algum treinamento relacionado a documentar, para fins legais, lesões resultantes da violência no lar? Que tipo de treinamento?

(8) Alguém perto de você - família, amigo ou colega - já experimentou violência doméstica?

(9) Você acredita que ter alguém próximo com uma história de violência doméstica afetaria a maneira como você lida com esses casos?
